# Approved drugs successfully repurposed against *Leishmania* based on machine learning predictions

**DOI:** 10.3389/fcimb.2024.1403589

**Published:** 2024-09-26

**Authors:** Rafeh Oualha, Yosser Zina Abdelkrim, Ikram Guizani, Emna Harigua-Souiai

**Affiliations:** Laboratory of Molecular Epidemiology and Experimental Pathology - LR16IPT04, Institut Pasteur de Tunis, Université de Tunis El Manar, Tunis, Tunisia

**Keywords:** drug repurposing, FDA-approved drugs, machine learning, *L. major*, *L. infantum*, *in vitro* validation, promastigotes, amastigotes

## Abstract

Drug repurposing is a promising approach towards the discovery of novel treatments against Neglected Tropical Diseases, such as Leishmaniases, presenting the advantage of reducing both costs and duration of the drug discovery process. In previous work, our group developed a Machine Learning pipeline for the repurposing of FDA-approved drugs against *Leishmania* parasites. The present study is focused on an *in vitro* validation of this approach by assessing the antileishmanial effects of 10 predicted drug candidates. First, we evaluated the drugs’ activity against promastigotes from two strains of *L. infantum* and one of *L. major*, which caused distinct clinical manifestations, using an MTT assay. The standard anti-*Leishmania* drug Amphotericin B was used as a positive control. Five molecules demonstrated anti-*Leishmania* effects, out of which Acebutolol, Prilocaine and Phenylephrine are described herein for the first time. When tested on promastigote growth, Acebutolol displayed IC_50_ values ranging from 69.28 to 145.53 µg/mL. Prilocaine exhibited IC_50_ values between 33.10 and 45.81 µg/mL. Phenylephrine, on the other hand, presented IC_50_ values >200 µg/mL. The two remaining drugs, Dibucaine and Domperidone, exhibited significantly low IC_50_ values varying between 0.58 and 1.05 µg/mL, and 6.30 and 8.17 µg/mL, respectively. Both compounds were previously described as anti-*Leishmania* agents *in vivo*. All five compounds demonstrated no notable cytotoxic effects on THP-1-derived macrophages at the IC_50_ concentrations, allowing for their testing on the intracellular form of *L. major* and *L. infantum* parasites. Interestingly, all compounds exhibited antileishmanial activity on amastigotes with enhanced IC_50_ values compared to the corresponding promastigotes. Noticeably, Dibucaine and Domperidone displayed IC_50_ values of at most 1.99 µg/mL. Acebutolol, Prilocaine and Phenylephrine showed IC_50_ values ranging from 13.84 to 66.81 µg/mL. Our previously published Computer-Aided repositioning pipelines of FDA-approved drugs as antileishmanial agents identified Dibucaine and Domperidone as candidates in support of previous *in vivo* studies. This study consolidates such findings through the *in vitro* validation against 2 *Leishmania* species, highly prevalent in Africa and Middle East, and reveals Acebutolol, Prilocaine, and Phenylephrine as novel anti-*Leishmania* effectors, confirming the relevance of our approach and calling for further investigations.

## Introduction

Leishmaniases are a group of widely distributed vector-borne diseases affecting 2M cases/year and causing more than 30,000 deaths annually. They are among the most neglected Tropical Diseases (NTDs) mostly affecting poor populations (World Health Organization (WHO), . The lack of low-cost and non-toxic treatments for these health conditions brings the urge to develop novel therapeutic strategies. However, the Research & Development (R&D) pipelines for drug discovery face many challenges that range from increasing costs and pressure on pricing to low success rates of discovery (Yildirim et al., 2016). This puts NTDs – including Leishmaniases – in the least important diseases for therapeutics development because of their poor financial potential in the market. An interesting avenue for proposing new drugs against NTDs with lower costs is drug repurposing, also called drug repositioning. This approach has the advantage of lowering the R&D process costs and minimizing withdrawal risks during the clinical trials (Ashburn and Thor, 2004; Charlton et al., 2018).

Drug repurposing has demonstrated its effectiveness in delivering novel applications of old drugs to treat NTDs, including Leishmaniases. Currently, the pentavalent antimonials represent the first line of antileishmanial drugs. They are commercially available as meglumine antimoniate (Glucantime^®^) or sodium stibogluconate (Pentostam^®^). Other drugs, mostly repurposed, are used as second-line treatments, namely: Amphotericin B, Pentamidine, Miltefosine and Paromomycin (Andrade-Neto et al., 2018; Santana et al., 2021; Altamura et al., 2022). Amphotericin B is an antifungal agent that was successfully repurposed against Leishmaniases but have demonstrated serious side effects (Andrade-Neto et al., 2018; Santana et al., 2021; Altamura et al., 2022). Its liposomal formulation (Ambisome) proved to be more effective and less toxic (Andrade-Neto et al., 2018; Santana et al., 2021; Altamura et al., 2022). Pentamidine, an aromatic diamidine, is used in the case of resistance to antimonials. However, its use has been limited due to its numerous side effects (Andrade-Neto et al., 2018; Santana et al., 2021; Altamura et al., 2022). Miltefosine, an anti-tumoral agent, is the first oral drug licensed for the treatment of Leishmaniases, thus giving access to home-based therapy. Nevertheless, it was also subject to multiple adverse effects such as toxicity, treatment failure, and the emergence of resistance (Andrade-Neto et al., 2018; Santana et al., 2021; Altamura et al., 2022). Stimaquine, an 8-aminoquinoline, is the second orally administered anti-leishmanial drug after Miltefosine. It presents multiple side effects such as nephrotoxicity, and demonstrates low efficacy (Loiseau et al., 2011).

Combination therapies for Leishmaniases are under investigation with the objective of increasing treatment efficacy and tolerance, reducing duration and cost, and limiting the emergence of drug resistance. Combination regimens associating Sodium stibogluconate and Paromomycin are currently integrated as first line treatment in visceral leishmaniasis. Other combination therapies are under the clinical phase investigation (DNDi, 2019; Santana et al., 2021; Altamura et al., 2022). Currently, a limited number of novel drug candidates have completed preclinical development and entered clinical trials such as DNDI-0690, DNDI-6148, DNDI-6899, DNDI-2319, GSK245 and LXE408 (DNDi, 2019; Santana et al., 2021; Altamura et al., 2022). Furthermore, there is currently no universal treatment for Leishmaniases and the efficacy of existing treatments may drop to as low as 50% (DNDi, 2020). Multiple research groups have also identified novel antileishmanial drugs candidates through clinical observations. This is especially the case for local anesthetics, like Lidocaine and Procaine, used during pre-clinical or clinical investigations prior to drug injections into the cutaneous lesions (Yépez and Scorza, 1995; Yépez et al., 1999). Observations of treatment enhancement associated with anesthetics application led to further investigation and validation of the antileishmanial effects (Cazorla Perfetti et al., 2004). In addition to clinical observations-based discoveries, multiple strategies were used for drug repurposing against Leishmaniases. We cite the phenotypic screening at medium to large scale (Fumarola et al., 2004) and the rational drug discovery approaches that focus on therapeutic targets of interest to identify potential inhibitors as lead compounds (Ding et al., 2011). Computational approaches hold high profiles in both approaches of drug discovery and repurposing. They present the advantages of enhancing the research outcomes, accelerating the process of discovery and reducing its costs (Baig et al., 2016). These methodologies involve molecular docking, protein-inhibitor interaction analysis, virtual screening, QSAR models and most recently artificial intelligence (AI) models (Desai et al., 2013; Harigua-Souiai et al., 2018, 2021; Chen et al., 2022). With the recent development in the AI field, computer-aided drug discovery witnessed a shift of paradigm. Machine learning (ML) and deep learning (DL) models have been successfully introduced into the drug discovery pipeline for multiple diseases, including leishmaniases. In the frame of leveraging the potential of ML models in drug discovery against Leishmaniases, our group has implemented a drug repurposing pipeline that proved to deliver valid predictions based on literature data review (Harigua-Souiai et al., 2022). Briefly, we have leveraged a large bioassay dataset of a biological screening of 65,057 chemical compounds against Leishmania major promastigote growth, resulting in 47,427 inactive compounds and 17,630 active compounds. We encoded the chemical structures of the molecules using multiple molecular fingerprints and assessed the accuracy of four machine learning algorithms in predicting the anti-Leishmania activity. The optimization of different components of the pipeline, including the algorithms, led to identifying Random Forest (RF) and Support Vector Machine (SVM) as the best classifiers. Thus we used both models to predict among the FDA approved drugs collection, those that can potentially be anti-Leishmania effectors. The pipeline delivered 19 potential active drugs against *Leishmania* with a confidence rate higher than 90%. Seven molecules appeared to be effectively active on the parasite based on literature review.

In the present work, we reported the experimental validation *in vitro* of 10 out of the 19 predicted molecules, including two that were previously *in vivo* tested according to the literature. Molecules were first tested *in vitro* for their inhibitory effects on the growth of extracellular parasites from one strain of *Leishmania major* and two strains of *Leishmania infantum*. Following this, active molecules were assessed for their cytotoxic effects on THP-1-derived macrophages, then tested on the intracellular form of *Leishmania major* and *L. infantum* parasites.

## Materials and methods

### Drugs and reagents

We purchased ten FDA approved drugs, previously predicted by our group through ML models, as potentially active against *Leishmania* parasites (Harigua-Souiai et al., 2022), namely Domperidone, Dibucaine, Phenylephrine, Acebutolol, Prilocaine, Albendazole, Ethacrynic acid, Ganciclovir, Benzthiazide and Ethionamide. The standard antileishmanial drug Amphotericin B was used as positive control. All compounds were commercially available in the Sigma-Aldrich (St. Louis, MO, USA) catalog except for Prilocaine and Ethacrynic acid, which were purchased from MedChemExpress. We listed all compound references, molecular weights and structures in the **Supplementary Material** (**Supplementary Table 1**). Stock solutions of all compounds were prepared as instructed by the manufacturer and kept in aliquots at -20°C. Then, intermediate concentrations of the selected compounds were freshly prepared in dimethyl sulfoxide (DMSO; Sigma-Aldrich) the day of the experiment.

### Parasites

Three Tunisian laboratory strains already described (Oualha et al., 2019), originally isolated from distinct clinical manifestations were used in this study: (i) *L*. *infantum* LV50 MHOM/TN/94/LV50) strain originating from a bone marrow of a visceral leishmaniasis patient, (ii) *L. infantum* Drep-14 (MHOM/TN/96/Drep-14) strain isolated from a lesion of a sporadic cutaneous leishmaniasis patient and (iii) *L. major* Empa-12 (MHOM/TN/2012/Empa-12) strain isolated from a zoonotic cutaneous leishmaniasis patient. Stocks of these parasites obtained after passage through BALB/c mice were used as previously described (Oualha et al., 2019). Thawed cryopreserved parasites were cultured at 22°C in RPMI-1640/Glutamax medium (Gibco BRL, Germany) containing penicillin (100 U/mL) and streptomycin (100 μg/mL) supplemented with 10% heat-inactivated Fetal Bovine Serum (FBS) (Gibco BRL, Germany). The growth kinetics of each strain were previously established and pinpointed the sixth day as part of the stationary growth phase.

### MTT-based promastigote viability assay

The effect of the selected compounds on the viability of Leishmania promastigotes was evaluated by a colorimetric MTT (3-(4,5-dimethylthiazol-2yl)-2,5-diphenyl tetrazolium bromide) assay. It consists in the reduction of tetrazolium salt to a soluble crystal (blue formazan) by the succinate dehydrogenase activity of mitochondria in living cells, which can be quantified by spectrophotometry. Briefly, 90 μL of promastigotes (5 × 10^5^ parasites/well) harvested from the stationary growth phase of each strain were added to a 96-well culture plate and incubated with 10 μL of a range of serial dilutions of the selected compounds in technical replicates (final concentration of 1% DMSO per well) (Ioset et al., 2009; Harigua-Souiai et al., 2018). The concentration range tested for each compound were as follows: (i) 3.12 to 200 µg/mL for Phenylephrine, Acebutolol, Ethacrynic acid, Ganciclovir and Ethionamide; (ii) 12.5 μg/mL to 300 μg/mL for Prilocaine; (iii) 1.56 µg/mL to 100 μg/mL for Domperidone and (iv) 0.23 μg/mL to 30 μg/mL for Dibucaine. Due to solubility issues, Benzthiazide and Albendazole were tested within the range of 0.78 to 50 µg/mL. After 72h of incubation at 22°C, 25 μl of MTT solution (5 mg/mL) was added to each well and incubated at 22°C for 4h. Then, 150 μl of DMSO was added to each well to dissolve the blue formazan and the optical density (OD) was measured at 570 nm with a microplate reader (MULTISCAN, Labsystems). Promastigotes incubated in complete medium containing 1% DMSO were used as negative control. The standard anti-Leishmania drug, Amphotericin B was used as a positive control. All experiments were performed in two technical replicates and repeated three times in independent experiments. The mean percentage of promastigotes viability was calculated from the Optical Density at 570 nm as follows:


$$\begin{array}{l} \text{Promastigote viability}\;(\%)=\;\\ {\left[\left(OD_{compound}-\;OD_{blank}\right)/\left(OD_{negative\;control\;}-OD_{blank}\right)\right]}_{*}100 \end{array}$$

Results were expressed as the mean value of the percentage of promastigotes viability ± standard deviation (SD). The concentration values corresponding to 50% of inhibition (IC_50_) were determined through fitting the dose-response curves of the logarithm of compound concentrations versus the % of promastigote viability for each compound to a nonlinear regression model, using GraphPad Prism version 8.0.1.

### Cytotoxicity assay towards THP-1-derived macrophages

FDA-approved drugs that exhibited an anti-Leishmania effect against the promastigotes were evaluated for their cytotoxic effect on THP-1-derived macrophages using the MTT assay as previously described (Harigua-Souiai et al., 2018; Abdelkrim et al., 2022). Briefly, THP-1 human monocyte cell line was grown in RPMI medium containing penicillin (100 U/mL) and streptomycin (100 μg/mL) with 10% fetal bovine serum (Gibco, Brazil) at 37°C in 5% CO_2_. To induce differentiation into adherent macrophages, THP-1 cells (5 × 10^4^) were seeded in 96-well plates and treated with 25 ng/mL of phorbol 12-myristate 13- acetate (PMA, Sigma, St. Louis, MO, USA). The PMA-treated THP-1 cells were maintained in this condition for 24 h, followed by a 24 h of rest period after the removal of PMA. Then, various concentrations of Acebutolol and Phenylephrine (3.13 to 200 µg/mL), Prilocaine (25 to 400 µg/mL), Dibucaine (0.47 to 30 µg/mL), Domperidone (1.56 to 100 µg/mL) or Amphotericin B (0.04 to 5 µg/mL) were added at a final concentration of 1% DMSO. Control cells were incubated in a supplemented RPMI medium containing 1% DMSO. The cytotoxicity towards THP-1-derived macrophages was assessed after 24, 48 and 72 hours of incubation by MTT assay as described above. The cytotoxicity of THP-1-derived macrophages was expressed as the percentage of the viable cells in treated cells relative to the THP-1-derived macrophages treated with 1% DMSO. The percentage of viable cells was calculated from the Optical Density at 570 nm as follows:


$$\begin{array}{l} \text{Cell viability}\;(\%)=\\ \;[\left(OD_{compound}-\;OD_{blank)}/\left(OD_{negative\;control}-\;OD_{blank}\right)\right]\;*\;100 \end{array}$$

The concentration causing a 50% reduction of viable macrophages (CC_50_) was determined by nonlinear regression of the logarithm of compound concentrations versus the % of cell viability from data of three independent experiments performed in two technical replicates using GraphPad Prism version 8.0.1.

### Anti-amastigote assay

THP-1-derived macrophages (5 × 10^4^/well) were seeded in each 8-well Labtek slides (Thermo Scientific). Adherent macrophages were obtained as described above, then infected with stationary phase promastigotes of each strain at a ratio 10:1 (10 parasites/macrophage) in RPMI medium containing penicillin (100 U/mL) and streptomycin (100 μg/mL) with 10% fetal bovine serum (Gibco, Brazil) at 37°C in 5% CO_2_ for 24 h. After infection, to ensure complete removal of extracellular promastigotes, infected cells were washed 4 times with PBS, and treated with increasing concentration of Acebutolol, Prilocaine and Phenylephrine (12.5, 25, 50, 100 and 200 µg/mL), Dibucaine (0.46, 0.93, 1.87, 3.75 and 7.5 µg/mL), Domperidone (0.78, 1.56, 3.12, 6.25 and 12.5 µg/mL) or Amphotericin B (0.03, 0.06, 0.125, 0.25 and 0.5 µg/mL). Non-infected control cells were incubated in a supplemented RPMI medium containing 1% DMSO. After 72h of incubation, cells were fixed and stained using May-Grunwald-Giemsa staining with RAL 555 Kit (RAL DIAGNOSTICS, France), following the manufacturer’s instructions. The percentage of infected cells and the total number of internalized parasites per 100 macrophages were determined under immersion oil (magnification of 1000X) using light microscopy. The percentage of amastigotes viability was expressed as the percentage of total number of internalized parasites within treated cells relative to the total number of internalized parasites within control cells treated with 1% DMSO, and calculated as follows:


Relative amastigote viability (%) = [(number of amastigotes/100 treated cells)/(number of amastigotes/100 treated cells with1% DMSO)] * 100

The relative percentage of amastigote viability was evaluated to calculate half inhibitory concentrations (IC_50_). The mean IC_50_ values were determined by plotting the curves of the logarithm of compound concentrations versus the relative amastigote viability of three independent experiments performed in two technical replicates for each compound fitted into a nonlinear regression model, using GraphPad Prism version 8.0.1. Selectivity Index (SI) values were calculated as the ratio between cytotoxicity and activity against amastigotes and determined for each compound as follows: SI= CC_50/_IC_50_.

### Statistical analysis

The data presented in this study corresponded to the Mean ± Standard Deviation (SD) of three independent experiments using GraphPad Prism 8.0.1. Student’s t-tests were used to assess the significance of differences between means. These differences were considered significant when the *p*-value was< 0.05.

## Results

### Ten FDA-approved drugs were selected for anti-*Leishmania* screenings

Based on previous work published by our group, a series of 19 FDA-approved drugs were predicted through two ML models to have a high probability of being anti-*Leishmania* candidates (Harigua-Souiai et al., 2022). Out of these, nine drugs were either commercially unavailable, extremely expensive for low quantities or previously characterized *in vitro* against multiple *Leishmania* species. As a final figure, we purchased the remaining ten molecules, out of which two were previously described through *in vivo* studies for their anti- *Leishmania* effects, namely Domperidone and Dibucaine (Cazorla Perfetti et al., 2004; Gómez-Ochoa et al., 2009; Passos et al., 2014; Cavalera et al., 2021). The eight remaining drugs, namely Phenylephrine, Acebutolol, Prilocaine, Albendazole, Ethacrynic acid, Ganciclovir, Benzthiazide and Ethionamide have not yet been described in the literature (**Table 1**). In **Table 1**, we summarized the original indication of each drug, its route of administration and the clinical dose used. The drugs were first screened for their antileishmanial activity *in vitro* against extracellular forms of *L. infantum* and *L. major* parasites. Active molecules were then tested for their cytotoxicity against macrophages and for their antileishmanial effects on intracellular forms of the same strains.

**Table 1:** List of FDA-approved drugs used in this study along their original indication, dose and route of administration.

Drug Name	Original indication	Dose*	RA*
Acebutolol	β1-receptor antagonist, used for the management of hypertension and ventricular premature beats	400 mg	Oral
Prilocaine	Local anesthetic used in dental procedures	40mg/mL	Dental/Topical
Phenylephrine	Alpha-1 adrenergic agonist used to treat acute hypotension	50 to 100 mcg	Intravenous
Dibucaine	Local anesthetic used for various medical purposes	Max 30g/24h	Topical
Domperidone	Dopamine receptor antagonist, treats nausea, vomiting, gastrointestinal problems like gastroparesis	10 mg	Oral
Albendazole	Benzimidazole antihelmintic used to treat parenchymal neurocysticercosis and other helminth infections	200 mg/Day	Oral
Ethacrynic acid	DNA polymerase inhibitor used to treat cytomegalovirus and herpetic keratitis of the eye	25 mg/Day	Oral
Benzthiazide	Is used to treat hypertension and edema	25 to 100 mg	Oral
Ethionamide	A second line antitubercular agent used to treat tuberculosis when other treatments have failed	250 mg to 500 mg/Day	Oral
Ganciclovir	A DNA polymerase inhibitor used to treat cytomegalovirus and herpetic keratitis of the eye	50 to 500 mg	Intravenous
Amphotericin B	An antifungal medication used to treat serious fungal infections, cryptococcal meningitis in HIV infection, and leishmaniasis	50 mg/ 10 mL	Intravenous

(*)Dose and Route of Administration (RA) correspond to those used for the pathology listed as the original indication of the drug. Information was mainly collected from the DrugBank website (Wishart et al., 2018).

### Five out of ten molecules demonstrated dose-response effects on promastigotes viability

**Figure 1:**
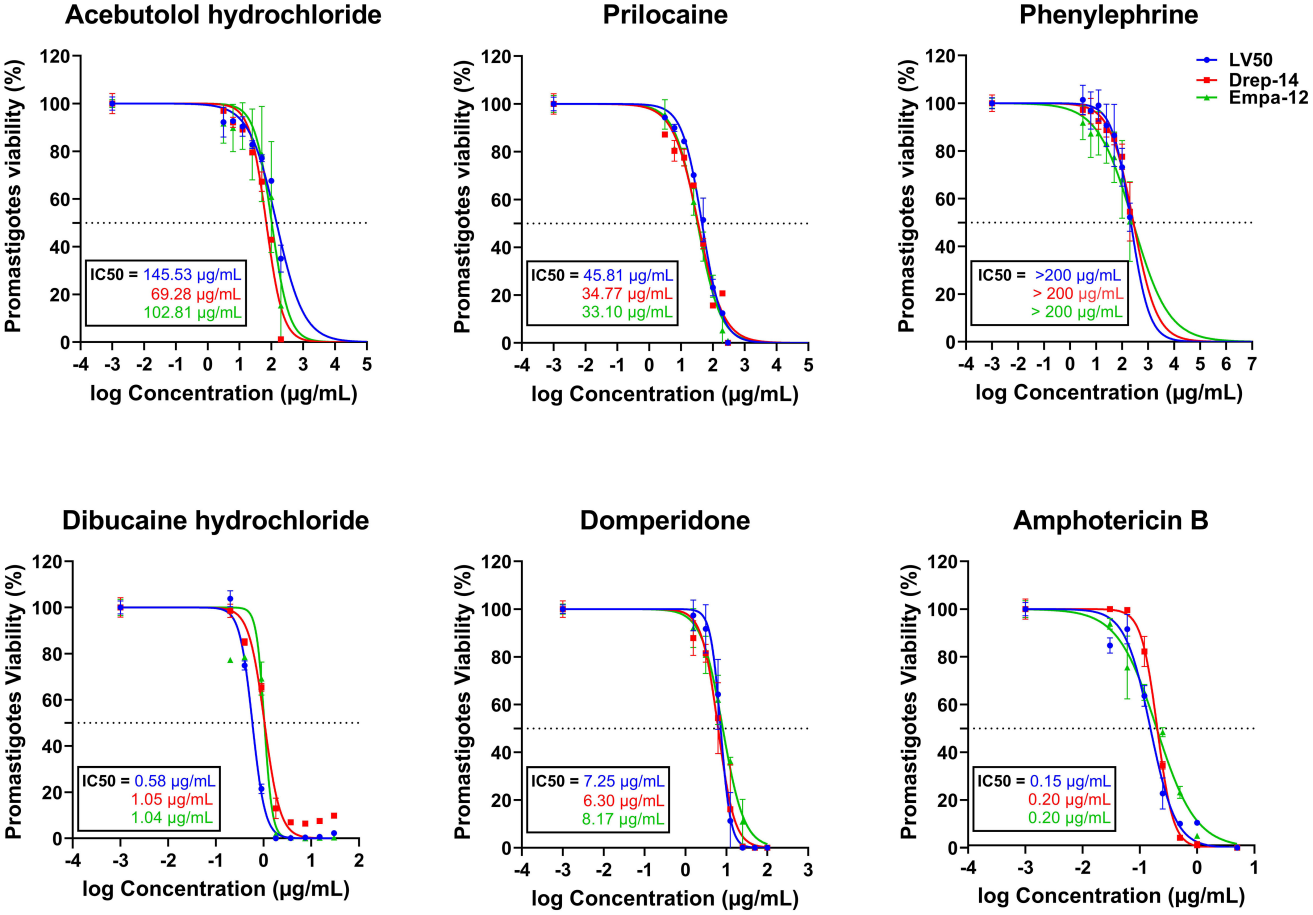
*In vitro* evaluation of the effect of five FDA-approved drugs against *L. infantum* (LV50 and Drep-14) and *L. major* (Empa-12) promastigotes. Promastigotes in the stationary growth phase were seeded in 96-well plates at a cell density of 5x10^5^ parasites/well and incubated with increasing concentrations of Acebutolol and Phenylephrine (3.12 to 200 µg/mL), Prilocaine (12.5 to 300 µg/mL), Dibucaine (0.2 to 30 µg/mL), Domperidone (1.56 to 100 µg/mL) or Amphotericin B (0.03 to 5 µg/mL). After 72h of incubation, parasite viability was evaluated with an MTT assay. The results were expressed as the percentage of promastigote viability treated with compounds relative to parasites treated with 1% DMSO. Data are shown as the mean values ± SD of three independent experiments carried out in technical duplicates.

We assessed the *in vitro* effects of ten FDA approved drugs (**Table 1**) selected through the ML-based approach (Harigua-Souiai et al., 2022), against *L. infantum* (LV50 and Drep-14) and *L. major* (Empa-12) promastigotes using an MTT assay. The standard anti-*Leishmania* drug, Amphotericin B, was used as a positive control. To this end, promastigotes in their stationary growth phase were seeded in 96-well plates (5 x 10^5^ parasites/well) and incubated for 72 h with increasing concentration of drugs as described in the Material and Methods section. Among the 10 compounds tested, five showed no effectiveness and did not impair parasite viability of the three strains LV50, Drep-14 and Empa-12, at the tested concentrations. These include, Albendazole, Ethacrynic acid, Ganciclovir, Benzthiazide and Ethionamide. The remaining five compounds, namely Domperidone, Dibucaine, Phenylephrine, Acebutolol and Prilocaine, presented dose-dependent decrease of parasite viability of all three strains (**Figure 1**), revealing an exceptional success rate of 50% for the present biological screening and the underlying ML-based approach (Harigua-Souiai et al., 2022).

Dibucaine presented the most significant effects on promastigote growth with IC_50_ values of 0.58 ± 0.02 µg/mL, 1.05 ± 0.06 µg/mL, and 1.04 ± 0.09 µg/mL on LV50, Drep-14 and Empa-12, respectively. Domperidone presented the second most interesting effects with IC_50_ values of 7.25 ± 0.26 µg/mL, 6.30 ± 0.38 µg/mL, and 8.17 ± 0.41 µg/mL, on LV50, Drep-14 and Empa-12, respectively (**Figure 1**). Acebutolol reduced parasite viability with IC_50_ values ranging between 145.53 ± 16.44 and 69.28 ± 5.70 µg/mL. Prilocaine affected parasite viability with IC_50_ values ranging between 33.10 ± 2.30 and 45.81 ± 2.46 µg/mL. Phenylephrine was the least potent overall and reduced promastigote viability to 50% at concentrations slightly higher than 200 µg/mL (**Figure 1**; **Table 2**). IC_50_ values of Phenylephrine on LV50, Drep-14 and Empa-12 were estimated to 218.59 ± 25.49, 275.28 ± 36.59 and 265.70 ± 79.54 µg/ml, respectively (**Supplementary Table 2**). The positive control Amphotericin B exhibited IC_50_ values ranging from 0.15 ± 0.01 to 0.20 ± 0.01 µg/mL, consistent with the literature findings (Kariyawasam et al., 2019).

**Table 2:** Summary of the *in vitro* effects of the FDA-approved drugs on the viability of the promastigotes, THP-1-derived macrophages, and intramacrophage amastigotes.

FDA approved drugs	Acebutolol	Prilocaine	Phenylephrine	Dibucaine	Domperidone	Amphotericin B
Promastigotes IC_50_ ( µg/mL)						
**LV50**	**145.53 ± 16.44** [390.28 ± 44.09 µM]	**45.81 ± 2.46** [207.93 ± 11.17 µM]	**> 200** [>1196.10 µM]	**0.58 ± 0.02** [1.53 ± 0.05 μM]	**7.25 ± 0.26** [17.00 ± 0.60 μM]	**0.15 ± 0.01** [0.16 ± 0.01 μM]
**Drep-14**	**69.28 ± 5.70** [185.79 ± 15.29 µM]	**34.77 ± 3.22** [157.82 ± 14.62 µM]	**> 200** [>1196.10 µM]	**1.05 ± 0.06** [2.76 ± 0.16 μM]	**6.30 ± 0.38** [14.80 ± 0.89 μM]	**0.20 ± 0.004** [0.22 ± 0.004 μM]
**Empa-12**	**102.81 ± 10.86** [275.71 ± 29.12 µM]	**33.10 ± 2.30** [150.24 ± 10.44 µM]	**> 200** [>1196.10 µM]	**1.04 ± 0.09** [2.74 ± 0.24 μM]	**8.17 ± 0.41** [19.20 ± 0.96 μM]	**0.20 ± 0.01** [0.22 ± 0.01 μM]
Cytotoxicity assays						
THP-1-derived macrophages* CC_50_ ( µg/mL)	**> 200** [>536.47 µM]	**> 400** [>1815.05 µM]	**> 200** [>1196.10 µM]	**22.7 ± 1.08** [59.74 ± 2.84 μM]	**26.5 ± 2.60** [62.40 ± 6.10 μM]	**> 5** [>5.41 µM]
Amastigotes IC_50_ ( µg/mL)						
**LV50**	**15.70 ± 2.75** [42.11 ± 7.38 µM]	**26.22 ± 3.14** [119.01 ± 14.25 µM]	**35.20 ± 4.86** [210.51 ± 29.06 µM]	**0.71 ± 0.09** [1.87 ± 0.24 μM]	**1.25 ± 0.15** [2.94 ± 0.35 μM]	**0.03 ± 0.004** [0.032 ± 0.004 μM]
**Drep-14**	**13.84 ± 1.41** [37.12 ± 3.78 µM]	**30.42 ± 2.30** [138.08 ± 10.44 µM]	**40.25 ± 3.15** [240.71 ± 18.83 µM]	**0.90 ± 0.03** [2.37 ± 0.08 μM]	**1.28 ± 0.09** [3.01 ± 0.21 μM]	**0.04 ± 0.006** [0.043 ± 0.005 μM]
**Empa-12**	**22.27 ± 1.59** [59.75 ± 4.27 µM]	**30.71 ± 2.20** [139.39 ± 9.99 µM]	**66.81 ± 7.28** [399.55 ± 43.53 µM]	**0.83 ± 0.05** [2.19 ± 0.13 μM]	**1.99 ± 0.10** [4.67 ± 0.23 μM]	**0.05 ± 0.005** [0.054 ± 0.005 μM]
Selectivity Index** (SI)						
**LV50**	**> 12.73**	**> 15.25**	**> 5.68**	**31.97**	**21.2**	**> 166.66**
**Drep-14**	**> 14.45**	**> 13.14**	**> 4.96**	**25.224**	**20.70**	**> 125.00**
**Empa-12**	**> 8.98**	**> 13.02**	**> 2.99**	**27.35**	**13.31**	**> 100.00**

IC_50_ values of the five drugs exhibiting activity against promastigotes and intracellular amastigotes of *L. major* and *L. infantum*. CC_50_ values of the five drugs on uninfected THP-1-derived macrophages, and their Selectivity Indexes (SI) computed based on the intracellular IC_50_ values. The mean of IC_50_ and CC_50_ values were determined from three independent experiments using Graphpad Prism.

(*)CC_50_ values correspond to those obtained after 72 h of treatment.

(**)Selectivity Index (SI) corresponds to the ratio between the CC_50_ and the IC_50_ values of intracellular amastigotes obtained after 72 h of treatment.

[]The values in brackets represent IC_50_ and CC_50_ values in µM. The values in bold represent IC50 and CC50 values in µg/mL, and SI values derived from them.

Noticeably, three out of the five active drugs are exclusively described for their anti-*Leishmania* effects in the present study, namely Phenylephrine, Acebutolol and Prilocaine. On the other hand, Dibucaine and Domperidone, previously described for potential anti-*Leishmania* effects in clinical settings, demonstrated promising inhibitory effects *in vitro* on the tested strains viability.

### Anti-*Leishmania* molecules presented no cytotoxic effects on the THP-1-derived macrophages at their IC_50_ concentrations

**Figure 2:**
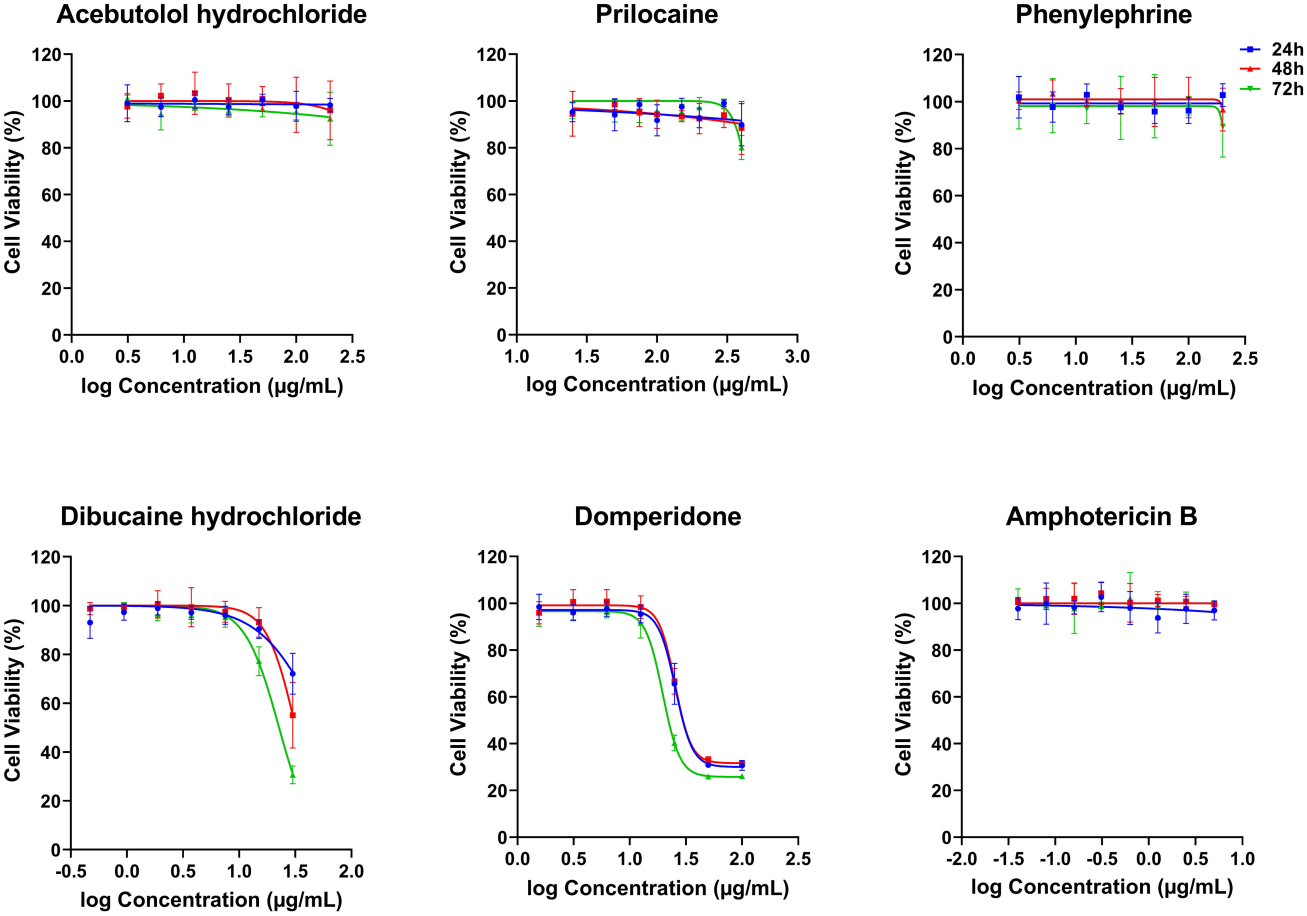
*In vitro* cytotoxicity effects of five FDA-approved drugs against THP-1-derived macrophages. THP-1-derived macrophages were incubated in the absence or presence of increasing concentrations of Acebutolol and Phenylephrine (3.13 to 200 µg/mL), Prilocaine (25 to 400 µg/mL), Dibucaine (0.47 to 30 µg/mL), Domperidone (1.56 to 100 µg/mL) or Amphotericin B (0.04 to 5 µg/mL). After 24, 48 and 72 hours of incubation, cytotoxicity towards THP-1-derived macrophages was evaluated using an MTT assay. The results were expressed as cell viability of THP-1-derived macrophages treated with compounds relative to the THP-1-derived macrophages treated with 1% DMSO. Data are shown as the mean values ± SD of three independent experiments carried out in technical duplicate.

The five compounds that exhibited anti-*Leishmania* effects were evaluated for their cytotoxic activity on THP-1-derived macrophages, at concentration ranges that include their respective IC_50_ values against the promastigotes. Interestingly, Acebutolol, Prilocaine and Phenylephrine showed no significant cytotoxicity on cells, even at high concentrations (**Figure 2**). Their respective CC_50_ values, after 72 h of incubation, greatly exceeded 200 µg/mL with 90-100% of macrophage viability observed at the same concentration (**Figure 2**; **Table 2**). For Prilocaine, we noticed a slight cytotoxic effect starting at a concentration of 400 µg/mL only in the case of 72 h of exposure, and interestingly 90-100% of macrophage viability at the corresponding IC_50_ value on the promastigotes (**Figure 2**). On the other hand, Dibucaine and Domperidone, exhibited CC_50_ values, after 72h of incubation of 22.70 ± 1.08 µg/mL and 26.50 ± 2.60 µg/mL, respectively (**Table 2**). Dibucaine started exhibiting cytotoxic effects at 30 µg/mL after 24 h and 48 h of incubation, and at 15 µg/mL after 72 h of incubation, and Domperidone showed detectable cytotoxic effects at 25 µg/mL under the three incubation times (**Figure 2**). Amphotericin B exhibited no cytotoxic effect on the cells, at concentrations up to 5 µg/mL. All CC_50_ values were summarized in **Table 2**. Taken together these results indicated that none of the tested molecules affected the viability of THP-1-derived macrophages at their IC_50_ values against promastigotes.

### Hits active on the extracellular parasites exhibited activity on the intracellular forms with enhanced IC_50_ values

The five compounds that demonstrated dose-dependent decrease of *Leishmania* promastigote viability were further assessed for their activity against the amastigote form of the three tested strains of *L. infantum* and *L. major*. Briefly, adherent macrophages (5 × 10^4^/well) were infected with stationary phase promastigotes of Empa-12, LV50 and Drep-14 at a ratio 10:1 for 24 h. Upon infection, THP-1 cell-derived macrophages were incubated for 72 h in the presence of increasing concentrations of the different compounds. Concentration ranges encompassed the respective IC_50_ values against the promastigotes for each compound and within the limit set by the cytotoxic concentrations on the THP-1-derived macrophages. The percentage of infected cells and the total number of internalized parasites per 100 macrophages were then counted. All active compounds against the *Leishmania* promastigotes proved to be effective against the amastigotes of all strains.

**Figure 3:**
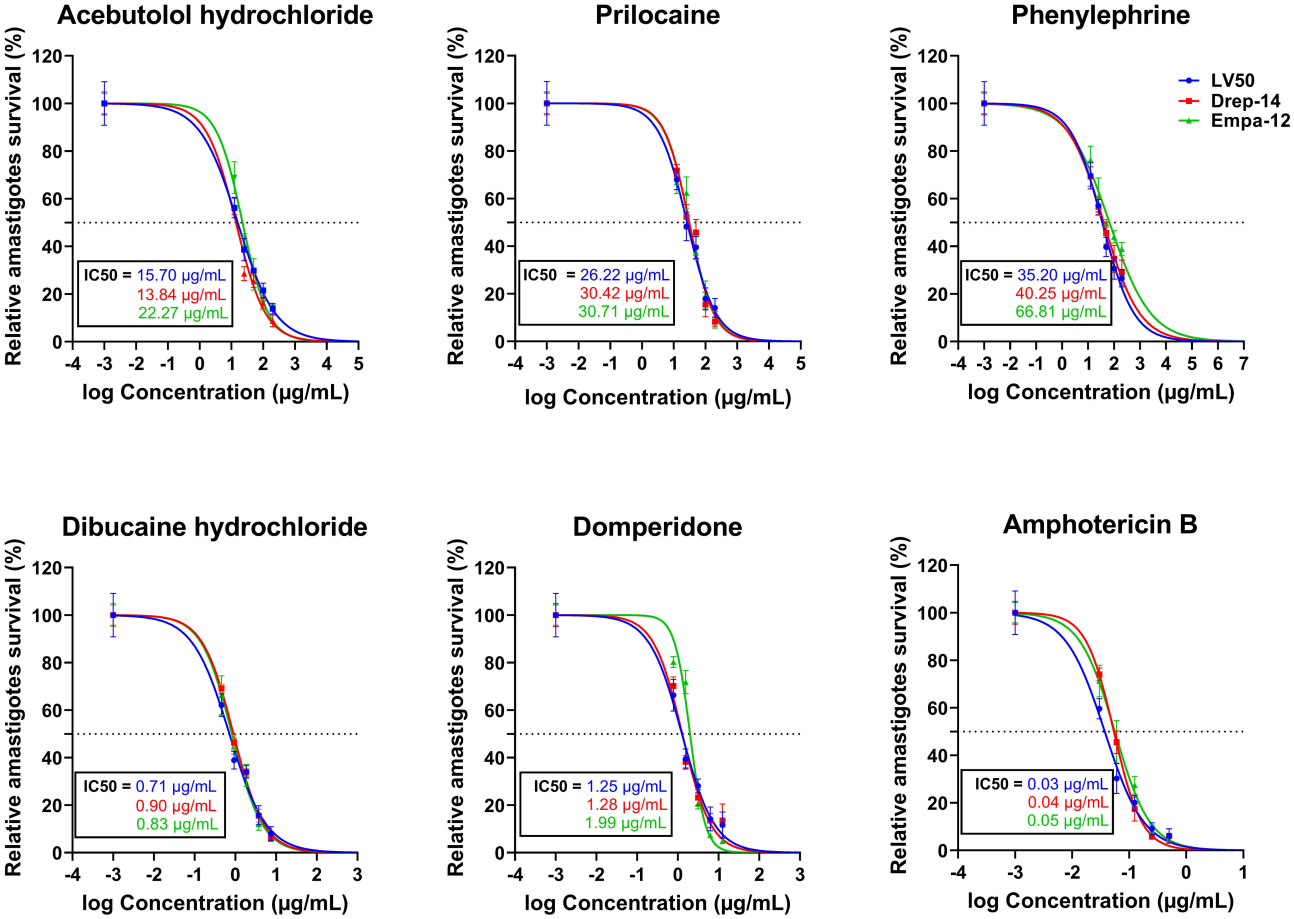
*In vitro* evaluation of the effect of five FDA-approved drugs against *L. major and L. infantum* amastigotes. THP-1-derived macrophages were infected with LV50, Drep-14 and Empa-12 strain for 24h, followed by a 72h incubation period in presence of increasing concentration of Acebutolol, Prilocaine and Phenylephrine (12.5 to 200 µg/mL), Dibucaine (0.46 to 7.5 µg/mL), Domperidone (0.78 to 12.5 µg/mL) or Amphotericin B (0.03 to 0.5 µg/mL). Cells were then fixed and stained with RAL 555 rapid stain kit. The total number of parasites per 100 infected macrophages treated with 1% DMSO or compounds was determined. The relative intracellular amastigote viability was expressed as the percentage of total number of parasites in treated cells relative to the total number of parasites in treated cells with 1% DMSO. Data are shown as the mean values ± SD of three independent experiments carried out in technical duplicates.

We could observe a dose-dependent decrease of the percentage of infected cells (**Supplementary Figure 1**) with increasing concentrations of all compounds. The percentage of relative amastigote viability was expressed as the percentage of total number of internalized parasites within treated cells relative to the total number of internalized parasites in control cells treated with 1% DMSO. The results demonstrated that 72 hours of exposure to all compounds led to a decrease of the percentage of amastigote viability, as shown in **Figure 3**. Based on these percentages, we calculated the IC_50_ values of each compound on the intracellular amastigotes. In accordance with the results obtained with the promastigotes, Dibucaine presented the most significant effect, with an IC_50_ value ranging from 0.71 ± 0.09 to 0.90 ± 0.03 µg/mL. Domperidone presented the second most interesting effect with an IC_50_ value ranging from 1.25 ± 0.15 to 1.99 ± 0.10 µg/mL. The IC_50_ values of Acebutolol ranged from 13.84 ± 1.41 to 22.27 ± 1.59 µg/mL. For Prilocaine, IC_50_ values ranged from 26.22 ± 3.14 to 30.71 ± 2.20 µg/mL and for Phenylephrine they ranged from 35.20 ± 4.86 to 66.81 ± 7.28 µg/mL. The positive control Amphotericin B exhibited IC_50_ values ranging from 0.03 ± 0.004 to 0.05 ± 0.005 µg/mL (**Figure 3**; **Table 2**). These results are consistent with previous studies, although variations may occur depending on the strain, species and experimental conditions (Tadele et al., 2019; Gedda et al., 2020). In summary, all five FDA-approved drugs that proved effective against extracellular parasites, along with Amphotericin B, demonstrated activity against the intracellular form, with enhanced IC_50_ values in the latter case.

In order to assess the prospectiveness of our hits, we calculated their selectivity indexes (SI). Phenylephrine was the least interesting compound, while Acebutolol and Prilocaine both exhibited moderate SI values. Domperidone and Dibucaine exhibited better SI values ranging from 21 to 32, except for the SI of Domperidone on Empa-12 (**Table 2**). Considering the fact that IC_50_ values for Domperidone and Dibucaine did not exceed 2 µg/mL, both compounds abide by the hit-to-lead selection criteria established by Nwaka and collaborators (Nwaka et al., 2009).

## Discussion

The present research falls within the context of identifying novel drug candidates against Leishmaniases through computer-aided drug repurposing. We aim at delivering promising lead compounds through a cost-effective approach that combines machine-learning methodologies to drug repurposing strategies for enhanced outcomes of the process, through reduced drop-out probabilities and costs. Our group previously published a series of FDA approved drugs that were predicted through two ML models to have high probability of being anti-*Leishmania* effectors (Harigua-Souiai et al., 2022). The present study is reporting experimental *in vitro* validation of ten drugs (out of the 19 previously predicted molecules) against the parasite in its extracellular and intracellular forms. Out of the ten molecules tested, five exhibited anti-*Leishmania* activity, namely Domperidone, Dibucaine, Prilocaine, Acebutolol and Phenylephrine.

Multiple groups have explored the effectiveness of Domperidone, a dopamine D2 receptor antagonist, in the treatment of visceral leishmaniasis in dogs (Gómez-Ochoa et al., 2009; Cavalera et al., 2021). In a clinical trial, the effects of Domperidone were assessed in 98 dogs naturally infected with *L. infantum*. During a 12-months follow-up, the dogs underwent clinical, serological, biochemical, and immunological tests. Results indicated that Domperidone effectively reduced clinical symptoms and antibody levels in diseased dogs, suggesting its high potential as a drug candidate to treat canine visceral leishmaniasis (Gómez-Ochoa et al., 2009). Another study demonstrated that Domperidone (Leisguard^®^) was able to improve serum creatinine and to reduce anti-*L. infantum* antibody titers, globulins, gamma globulins, and C-reactive protein (CRP) in dogs affected with leishmaniasis and chronic kidney disease (Cavalera et al., 2021). The therapeutic effect of Domperidone in dogs has been attributed mainly to its immunomodulatory activity shown in other studies (Passos et al., 2014; Travi and Miró, 2018). Most of these studies (Gómez-Ochoa et al., 2009; Cavalera et al., 2021; Travi and Miró, 2018; Passos et al., 2014) have mainly addressed the immune response using non-invasive sampling methods to conduct serological, biochemical and immunological examinations. Therefore, the impact of treatment on canine VL cases with Domperidone on parasite burden in target organs (spleen, bone marrow, lymph nodes and/or skin) was not determined. To the best of our knowledge, no experiments addressed the direct effect of a Domperidone-based treatment on parasite burden in target organs nor on lesion healing in animal models. Nevertheless, aside from the present work, a single *in vitro* study assessed Domperidone’s efficacy against both promastigotes and amastigotes of *L. amazonensis* (Andrade-Neto et al., 2016). Although Domperidone displayed activity against promastigotes with an IC_50_ value of 51 µM (10.6 µg/mL), its IC_50_ value against amastigotes was lower than the established threshold of 25 μM (5.2 µg/µL) (Andrade-Neto et al., 2016). In our study we confirmed this observation on the promastigote form of *L. infantum* and *L. major* with IC_50_ values ranging from 6.30 to 8.17 µg/mL. The IC_50_ values against intracellular amastigotes ranged from 1.25 to 1.99 µg/mL. In conclusion, there is a need to further validate the direct effect of this drug on parasite burden in animal models, and thus establish the direct correlation between previously observed effects of Domperidone on canine VL and CL cases and the results herein obtained through *in vitro* models. Additional research would also unveil the mechanism of action and the molecular targets in *Leishmania* parasites, as they do not have an ortholog of the dopamine D2 receptor found in higher eukaryotes. The administration of dopamine receptor antagonists may cause undesirable physiological effects, highlighting the need for further comprehensive safety studies towards identifying safe effective doses.

Direct testing *in vivo* appeared as a time-saving approach for multiple groups working on drug discovery. In this context, Dibucaine was directly tested *in vivo*, on hamsters (Cazorla et al., 2004). Based on the literature data reporting successful trials conducted on Lidocaine, these authors considered assessing two other “cainic” local anesthetics, namely Procaine and Dibucaine, for their anti-*Leishmania* effects. The study revealed that an intralesional treatment with Dibucaine was comparable to the standard dosages of Glucantime^®^, while Procaine demonstrated lower efficacy (Cazorla et al., 2004). Local treatment with Dibucaine was as clinically efficient as systemic Glucantime^®^ and was more successful in reducing the size of the lesions in hamsters experimentally infected with *Leishmania (Viannia) braziliensis* as compared to Procaine (Cazorla et al., 2004). Interestingly, and to the best of our knowledge, the present work is the first report on the anti-leishmanial *in vitro* activity of Dibucaine. It effectively killed *in vitro* the extracellular parasites of both *L. major* and *L. infantum*, with IC_50_ values ranging from 0.58 µg/mL to 1.05 µg/mL. It exhibited comparable IC_50_ on the intracellular forms and promising selectivity indexes (>25) that supported its potential as a treatment option against leishmaniases (Nwaka et al., 2009).

Prilocaine, another “cainic” local anesthetic pharmacologically similar to Lidocaine, is used in dental procedures. It acts by targeting sodium channels on the neuronal cell membrane, limiting the spread of seizure activity, and reducing seizure propagation. As opposed to other anesthetics such as Lidocaine, Procaine, and Dibucaine, previously reported for their anti-*Leishmania* activity, the effect of Prilocaine on *Leishmania* parasites *in vitro* is originally revealed in the present work. Moreover, a wide spectrum of its inhibitory effect has been shown on various microorganisms, including *E. coli*, *S. aureus*, *P. aeruginosa* (Aydin et al., 2001). Thus, the efficacy of prilocaine as a local anesthetic, along with its anti-leishmanial properties, could potentially be of interest as a topical treatment candidate against cutaneous leishmaniasis, or as part of combination therapy despite its moderate IC_50_ values *in vitro*. Similarly, Fluconazole, an anti-*Leishmania* treatment, exhibited high IC_50_ values *in vitro* superior to 256 and 275 µg/mL against different strains and species (Plourde et al., 2012; Kariyawasam et al., 2019), and estimated to 140 ± 50µM against multiple strains of *L. donovani* (Pandharkar et al., 2014). This is also the case for first-line treatments for leishmaniasis, such as sodium stibogluconate (Pentostam), which exhibited IC_50_ values against *L. tropica* strains ranging from 142 to 300 µg/mL, *i.e.* 155 to 309µM (Plourde et al., 2012).

In the present work, we also report on the original anti-*Leishmania* effects of two additional FDA-approved drugs, namely Acebutolol and Phenylephrine. Both drugs exhibited high IC_50_ values on both forms of *Leishmania* parasites with low cytotoxic effects on the THP-1-derived macrophages. In high eukaryotes, Phenylephrine and Acebutolol both interact with the adrenergic receptor system. Phenylephrine is specifically classified as a selective α1-adrenergic receptor agonist that primarily increases blood pressure by raising systemic vascular resistance (Wishart et al., 2018), while Acebutolol is a selective ß1-receptor antagonist used in managing cardiovascular conditions such as hypertension, angina, and heart rhythm abnormalities (Wishart et al., 2018). The anti-*Leishmania* effects of other adrenergic agonists and antagonists, such as propranolol, epinephrine, isoproterenol, and dibutyryl cAMP (db-cAMP), have been reported when tested at 100 µM for 96 hours, exhibiting 52% to 95% growth inhibition compared to the control group (Kar and Kar, 2018). These findings should be approached cautiously due to the dose limits that can be administered without affecting adrenergic receptors.

Strategically, our ML based approach not only has the capacity to shorten treatment duration and reduce costs but may also play a crucial role in mitigating risks associated with drug resistance while minimizing side effects. In fact, the route of administration is a major drawback for some of the currently available drugs for Leishmaniases. This study has identified five potential drug candidates, some of which require further investigation including studies of the mechanism of action and molecular targets in *Leishmania* parasites, comprehensive safety studies, with promising differential potential through *in vivo* and preclinical studies. Among these candidates, Dibucaine and Prilocaine are anesthetics available in topical formulation (**Table 1**). This presents a favorable option for cutaneous manifestations of the disease, as it allows for localized treatment while potentially reducing systemic side effects, accelerating treatment options for cutaneous Leishmaniases, the most neglected among the NTDs. On the other hand, among the three remaining candidates, Domperidone can be administered orally (**Table 1**), enhancing its convenience and ease of use. Topical or oral formulations are highly relevant especially in areas with limited access to medical care and advanced infrastructure.

## Data Availability

The original contributions presented in the study are included in the article/**Supplementary Material**, further inquiries can be directed to the corresponding author/s.
