# Supplementary material for: Approved drugs successfully repurposed against Leishmania based on machine learning predictions
Source: Front Cell Infect Microbiol. 2024 Sep 26;14:1403589. doi: 10.3389/fcimb.2024.1403589 (PMC11464777; doi:10.3389/fcimb.2024.1403589)
Supplement: Supplementary file 1 [file DataSheet1.pdf]

Supplementary Material

Approved Drugs Successfully repurposed against *Leishmania* Based on Machine Learning Predictions

Rafeh Oualha[1], Yosser Zina Abdelkrim[1], Ikram Guizani[1], Emna Harigua-Souiai [1*]

*** Correspondence: Emna Harigua-Souiai**: emna.harigua@pasteur.utm.tn

1 Supplementary Figures and Tables

1.1 Supplementary Figure 1: *In vitro* evaluation of the effect of FDA-approved Drugs on the percentage of infected THP-1 cells

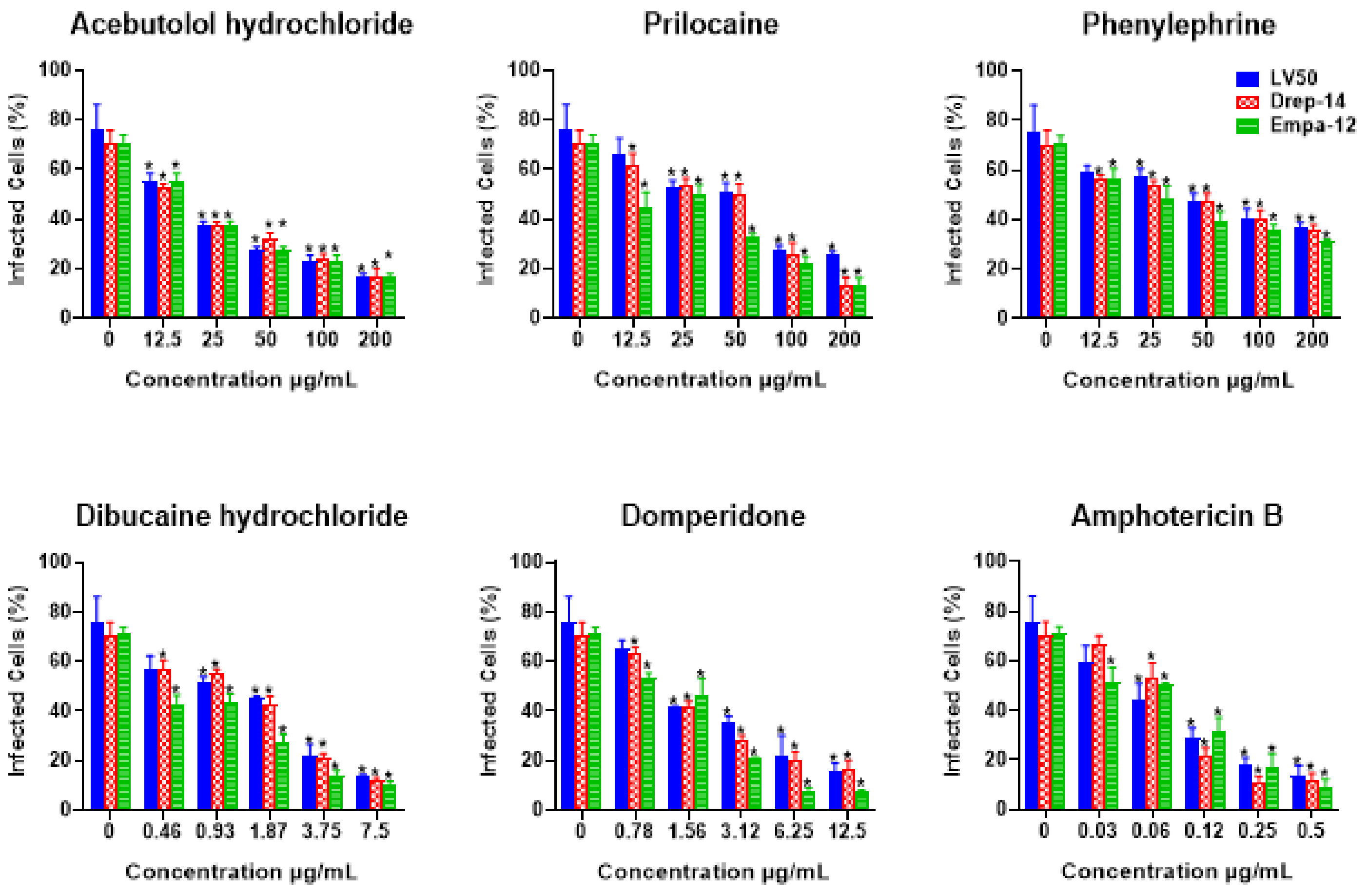

Supplementary Material

Supplementary Figure 1. *In vitro* evaluation of the effect of FDA-approved Drugs on the percentage of infected THP-1 cells. THP-1-derived macrophages were infected with Empa-12 strain at a ratio 10:1 (10 parasites/macrophage) in RPMI containing 10% fetal bovine serum at 37°C in 5% CO_2 for 24 h. After infection, to ensure complete removal of extracellular promastigotes, infected cells were washed 4 times with PBS, and treated with increasing concentration of Acebutolol, Prilocaine, and Phenylephrine (12.5, 25, 50, 100 and 200 µg/mL), Dibucaine (0.46, 0.93, 1.87, 3.75 and 7.5 µg/mL), Domperidone (0.78, 1.56, 3.12, 6.25 and 12.5 µg/mL) or Amphotericin B (0.03, 0.06, 0.12, 0.25 and 0.5 µg/mL). Infected control cells were incubated in supplemented RPMI medium containing 1% DMSO. After 72 h of incubation, cells were fixed and stained with RAL 555 rapid stain kit. The percentage of infected cells were determined under immersion oil (magnification of 1000X) using light microscopy. The results were expressed as the percentage of treated infected cells relative to the untreated cells. Data are shown as the mean values ± SD of three independent experiments carried out in technical duplicate. *$p < 0.05$ indicates statistically significant differences.

1.2 Supplementary Table 1: List, references and structures of FDA-approved Drugs used in this study

Drug Name	Reference*	Structure
Acebutolol hydrochloride	A3669	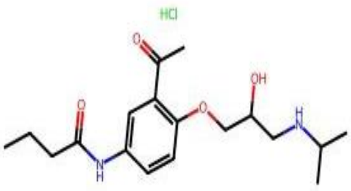
Prilocaine	HY-B0137/<br>CS-1929	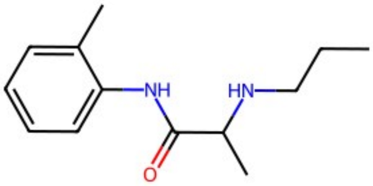
Phenylephrine	P1240000	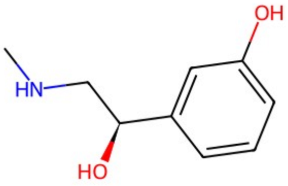

Dibucaine hydrochloride	D0638	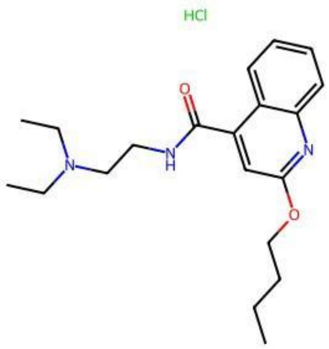
Domperidone	D122	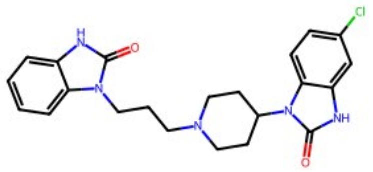
Albendazole	A4673	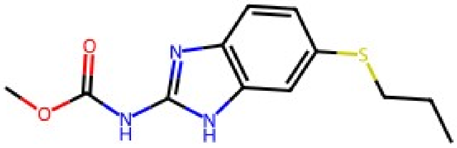
Ethacrynic acid	HY-B1640	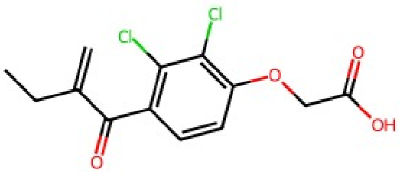
Benzthiazide	B7149	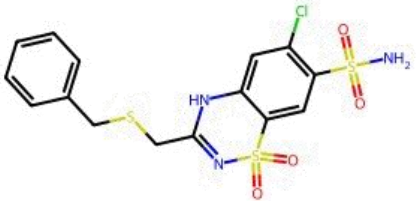
Ethionamide	E6005	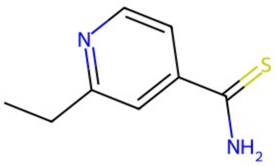

Ganciclovir	G2536	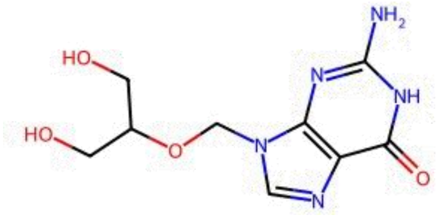
Amphotericin B	A2942	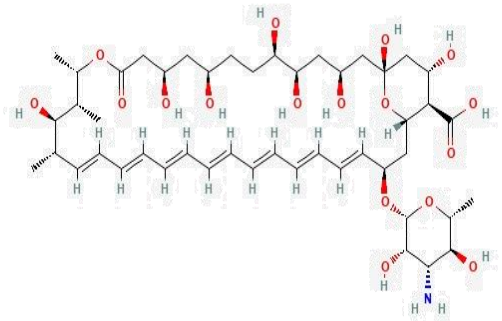

(*) All compounds were commercially available in the Sigma-Aldrich (St. Loui, MO, USA) catalog except for Prilocaine and Ethacrynic acid, which were purchased from MedChemExpress**.**

1.3 Supplementary Table 2: IC50 values (µg/mL) of Phenylephrine estimated through extrapolation, using GraphPad version 8.0.1

**Phenylephrine**	**LV50**	**Drep-14**	**Empa-12**
**IC50 (µg/mL)**	218.59 ± 25.49	275.28 ± 36.59	265.70 ± 79.54
